# Emergency obstetric and newborn care signal functions in public and private facilities in Bangladesh

**DOI:** 10.1371/journal.pone.0187238

**Published:** 2017-11-01

**Authors:** Lumbini Roy, Taposh Kumar Biswas, Mahbub Elahi Chowdhury

**Affiliations:** 1 Health Systems and Population Studies Division, International Centre for Diarrhoeal Disease Research, Bangladesh (icddr,b), Dhaka, Bangladesh; 2 Maternal and Child Health Division, International Centre for Diarrhoeal Disease Research, Bangladesh (icddr,b), Dhaka, Bangladesh; National Academy of Medical Sciences, NEPAL

## Abstract

**Background:**

Signal functions for emergency obstetric and newborn care (EmONC) are the major interventions for averting maternal and neonatal mortalities. Readiness of the facilities is essential to provide all the basic and comprehensive signal functions for EmONC to ensure emergency services from the designated facilities. The study assessed population coverage and availability of EmONC services in public and private facilities in Bangladesh.

**Methods:**

An assessment was conducted in all the public and private facilities providing obstetric care in to in-patients 24 districts. Data were collected on the performance of signal functions for EmONC from the study facilities in the last three months prior to the date of assessment. Trained data-collectors interviewed the facility managers and key service providers, along with review of records, using contextualized tools. Population coverage of signal functions was assessed by estimating the number of facilities providing the signal functions for EmONC compared to the United Nations requirements. Availability was assessed in terms of the proportion of facilities providing the services by type of facilities and by district.

**Results:**

Caesarean section (CS) delivery and blood transfusion (BT) services (the two major components of comprehensive EmONC) were respectively available in 6.4 (0.9 public and 5.5 private) and 5.6 (1.3 public and 4.3 private) facilities per 500,000 population. The signal functions for basic EmONC, except two (parental anticonvulsants and assisted vaginal delivery), were available in a minimum of 5 facilities (public and private sectors combined) per 500,000 population. A major inter-district variation in the availability of signal functions was observed in each public- and private-sector facility. Among the various types of facilities, only the public medical college hospitals had all the signal functions. The situation was poor in other public facilities at the district and sub-district levels as well as in private facilities.

**Conclusions:**

In the public sector, CS delivery and BT services were available in the minimum required number of facilities. However, to ensure basic EmONC services, participation of the private sector is necessary. Public-private partnership should be promoted for nationwide coverage of signal functions for EmONC in Bangladesh.

## Introduction

Globally, more than half of the maternal deaths result from haemorrhage, hypertensive disorders, and sepsis [1]. These are preventable by a few medical interventions termed `signal functions’ for emergency obstetric and newborn care (EmONC) defined by the United Nations (UN) [2,3]. A designated comprehensive EmONC (CEmONC) facility should have nine specific signal functions, such as (i) administering parenteral antibiotics, (ii) administering uterogenic drugs for active management of the third stage of labour and prevention of postpartum haemorrhage, (iii) use of parenteral anticonvulsants for the management of pre-eclampsia/eclampsia, (iv) manual removal of placenta, (v) removal of retained products (e.g. manual vacuum extraction, dilatation, and curettage), (vi) performing assisted vaginal delivery (AVD), i.e. vacuum extraction or forceps delivery, (vii) performing basic neonatal resuscitation), (viii) performing CS delivery, and (ix) BT services to be available for 24 hours a day, 7 days a week [3,4]. A designated basic EmONC (BEmONC) facility should have seven signal functions, excluding CS delivery and BT services [3].

Maternal mortality ratio has decreased remarkably in many South Asian countries, including Sri Lanka, Bhutan, and Maldives [5]. Although maternal mortality ratio (MMR) in Bangladesh has reduced by 40% in the last decades [6], it still remains higher than that in the countries mentioned above. The major causes of maternal deaths in Bangladesh are haemorrhage and eclampsia/preeclampsia [5] that are essentially required to be managed at health facilities with relevant signal functions. For instance, haemorrhage can be compensated for BT; during administering parenteral oxytocin, manual removal of placenta, and removal of retained products, timely BT may be necessary in the event of any haemorrhage. Administering parenteral anticonvulsants is essential for the management of eclampsia.

The UN also recommends that there should be at least 5 EmONC facilities per 500,000 population, of which at least one should provide all the signal functions of CEmONC [3,4]. However, just having adequate number of facilities does not necessarily ensure the availability of service. Actual functionality of any designated EmONC facility with the signal functions is important. Therefore, population coverage of the signal functions for EmONC is considered an important indicator for preventing maternal and perinatal deaths. Bangladesh has a good healthcare service-delivery network from the national level to the community level. To improve the accessibility of services by the community people, initiative has been taken to make the services available at all levels of the healthcare delivery system. To make healthcare equitable and accessible by the poor in Bangladesh, the public facilities are providing healthcare free of charge. Thus, the Government has designated different hospitals at the upazila (sub-district) to the national level as EmONC facilities to increase the population coverage according to the UN definition. However, there is a concern about the availability of services in those designated EmONC facilities. Relatively higher rates of child delivery (17% vs 12%) and CS delivery (8% vs 3.5%) in the private-sector facilities than in the public-sector facilities in Bangladesh [7] also support the evidence of unavailability of the required EmONC services in the designated public facilities. In addition, the disparity between the poorest and the richest segment of population for facility-based delivery (1:6) and CS delivery (1:15) is also alarming [7]. To address these inequalities, the public facilities need to be functional to make the services accessible to the poor. Therefore, there is a need to identify the existing gaps in providing EmONC services from the public facilities, along with population coverage in order to ensure availability of signal functions. Moreover, the huge expansion of the private sector in Bangladesh [8] demands considerable attention as it leads to opportunities in filling-up the gaps in service provision to strengthen the capacity of the health systems run by public-private-partnership (PPP). Therefore, the current study was undertaken to determine the gaps in population coverage and availability of signal functions for EmONC in designated facilities of both public and the private sectors.

## Materials and methods

The wording of the manuscript is suitable for publication.

### Study design

**Fig 1:**
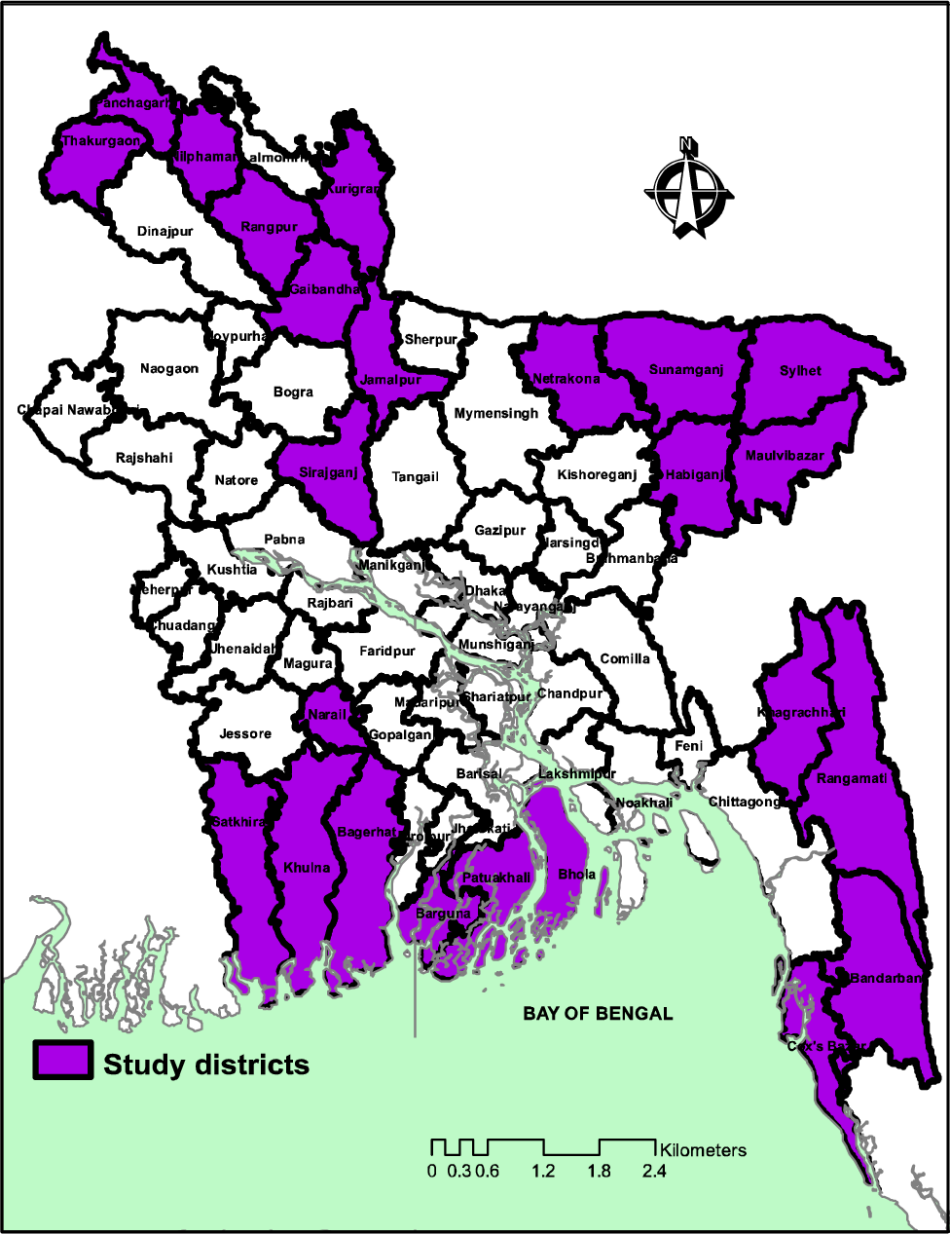
The 24 study districts in Bangladesh.

A cross-sectional facility assessment was conducted during May-October 2012 (Fig 1).

### Study settings and population

The study was conducted in 24 low-performing districts of Bangladesh, based-on the poor health indicators of MDGs as identified by the United Nations Development Assistance Framework [9] and Maternal and Neonatal Health Intervention Program of the Government [10]. Due to difficult geographic terrain, these are comparatively hard-to-reach areas that need special attention for improving the health indicators. The study included all the public facilities at the district and sub-district levels and the private not-for-profit (nfp) and for-profit (fp) facilities providing maternal and newborn health (MNH) care services to in-patients.

Public facilities included medical college hospitals (MCHs), district hospitals (DHs), mother and child welfare centres (MCWCs), upazilla health complexes (UHCs), and other public in-patient hospitals (IPHs). All MCHs and DHs were designated as CEmONC facilities; MCWCs and UHCs were categorized as either CEmONC or BEmONC facilities based on their status. In the case of private nfp and fp facilities, those that provided delivery care and had at least one bed for in-patient were included in the assessment. In total, 800 facilities of various types in both public and private sectors at different tiers of the health system were included in the study (**Table 1**).

**Table 1:** Number of facilities by type, assessed for availability of signal functions for EmONC in the 24 study districts of Bangladesh.

Type of facility		Number of facility
Public facilities designated for CEmONC	Medical college hospital	3
	District hospital	23
	Mother & child welfare centre	26
	Upazila health complex	53
Public facilities designated for BEmONC	Mother & child welfare centre	9
	Upazila health complex	105
Other public facilities	Other public in-patient hospital	15
Private facilities	Private not-for-profit facility	89
	Private for-profit facility	477
Total		800

### Methods of data collection

A provisional list of facilities offering MNH care services was collected from the Ministry of Health and Family Welfare of Bangladesh. This list was further updated by comparing with the lists that existed in the offices of the civil surgeons and the deputy directors of family planning in each district. With these updated lists of facilities, trained interviewers visited the public facilities designated for EmONC services and identified private (both nfp and fp) facilities offering obstetric and newborn care services in the respective districts and sub-districts.

We used contextualized tools for EmONC needs assessment that was originally developed by the Averting Maternal Death and Disability (AMDD) group based in the Columbia University, USA. The tools were contextualized for different types of health facilities in Bangladesh through a joint effort by the members of a core team involving co-investigators of the study, including an Obs/Gyn specialist and key members of the AMDD group.

In total, 6 teams were assigned to collect data from the district and subdistrict levels, each consisting of 4 members headed by one medical officer (MO). The field teams were thoroughly trained on ethical and quality issues relating to data collection. Upon taking informed written consent from the head of each facility, one health manager and service providers involved in EmONC were interviewed to assess the availability of signal functions. The signal functions were considered available if the respective services were provided within the last three months prior to the date of assessment. The care providers’ verbal information was further verified cross-checking against records in the registers and was considered final. All the collected data were reviewed at the end of each day by the supervisor and, for any inconsistency, the health facilities were revisited for necessary corrections.

### Data analysis

The data were computerized using a customized data-entry application package with the provision of checking for errors. The data were managed in a relational database management system (SQL server) maintaining the linkage among health facilities at different hierarchical levels of the health system. All the entered data were further checked for internal inconsistency by applying logical conditions. For any internal inconsistency, necessary corrective measures were taken after comparing the entered data with those in the questionnaire.

Analysis was done following the “Needs Assessment of Emergency Obstetric and Newborn Care: Data Analysis Guide” of AMDD [11]. Population coverage of signal functions was estimated following UN guideline by calculating the number of facilities for 500,000 population, providing related services in the last three months prior to the date of assessment using the population census data of 2011 as the denominators [12]. For the availability of signal functions, we calculated the percentage of facilities by type of services provided.

### Ethics statement

Ethical approval to conduct the study was obtained from the Ethics Review Committee of International Centre for Diarrhoeal Disease Research, Bangladesh (icddr,b). Informed written consent was taken from the head of each selected facility before conducting the assessment.

## Results

Overall, the public sector alone did not have adequate coverage of the availability of signal functions for EmONC in the study districts. However, the public and private sectors together could substantially improve the coverage of most signal functions (Table 2). In the public sector, though the CS delivery and BT services were available in about 0.9 and 1.4 public facilities per 500,000 populations respectively, for the rest 7 signal functions, the service availability was only in 0.4 to 2.4 facilities per 500,000 population. However, the aggregated data from the public and private facilities showed that 7 out of 9 signal functions (except administering of parenteral anticonvulsants and AVD), had adequate service coverage i.e. at least 5 facilities per 500,000 population had the signal function.

**Table 2:** Population coverage of signal functions for EmONC in the last 3 months prior to the date of assessment in the 24 study districts of Bangladesh.

Signal functions	Number of facilities (no. per 500,000 population) having signal functions					
	Public facilities designated for CEmONC	Public facilities designated for BEmONC	Other public IPHs	Private nfp facilities	Private fp facilities	All
**Caesarean section**	70 (0.80)	9 (0.10)	1(0.01)	37 (0.42)	445 (5.10)	565 (6.43)
**Blood transfusion**	69 (0.79)	34 (0.39)	5(0.05)	31 (0.35)	342 (3.92)	477 (5.50)
**Parenteral antibiotics**	104 (1.19)	104 (1.19)	9(0.10)	65 (0.74)	450 (5.15)	730 (8.37)
**Parenteral oxytocics**	105 (1.20)	99 (1.13)	5(0.06)	73 (0.84)	446 (5.11)	728 (8.34)
**Parenteral anticonvulsants**	70 (0.80)	53 (0.61)	2(0.02)	27 (0.31)	223 (2.55)	375 (4.29)
**Manual removal of placenta**	87 (1.00)	72 (0.82)	4(0.05)	44 (0.50)	275 (3.15)	482 (5.52)
**Removal of retained products**	82 (0.94)	62 (0.71)	5(0.06)	31 (0.35)	330 (3.78)	510 (5.84)
**Assisted vaginal delivery**	24 (0.27)	9 (0.10)	0(0.00)	5 (0.06)	31 (0.35)	69 (0.78)
**Neonatal resuscitation**	85 (0.97)	70 (0.80)	3(0.03)	44 (0.50)	258 (2.95)	457 (5.25)

EmONC = Emergency Obstetric and Newborn Care; CEmONC = Comprehensive Emergency Obstetric and Newborn Care; BEmONC = Basic Emergency Obstetric and Newborn Care; IPHs = In-patient hospitals; nfp = not-for-profit; fp = for-profit

**Fig 2:**
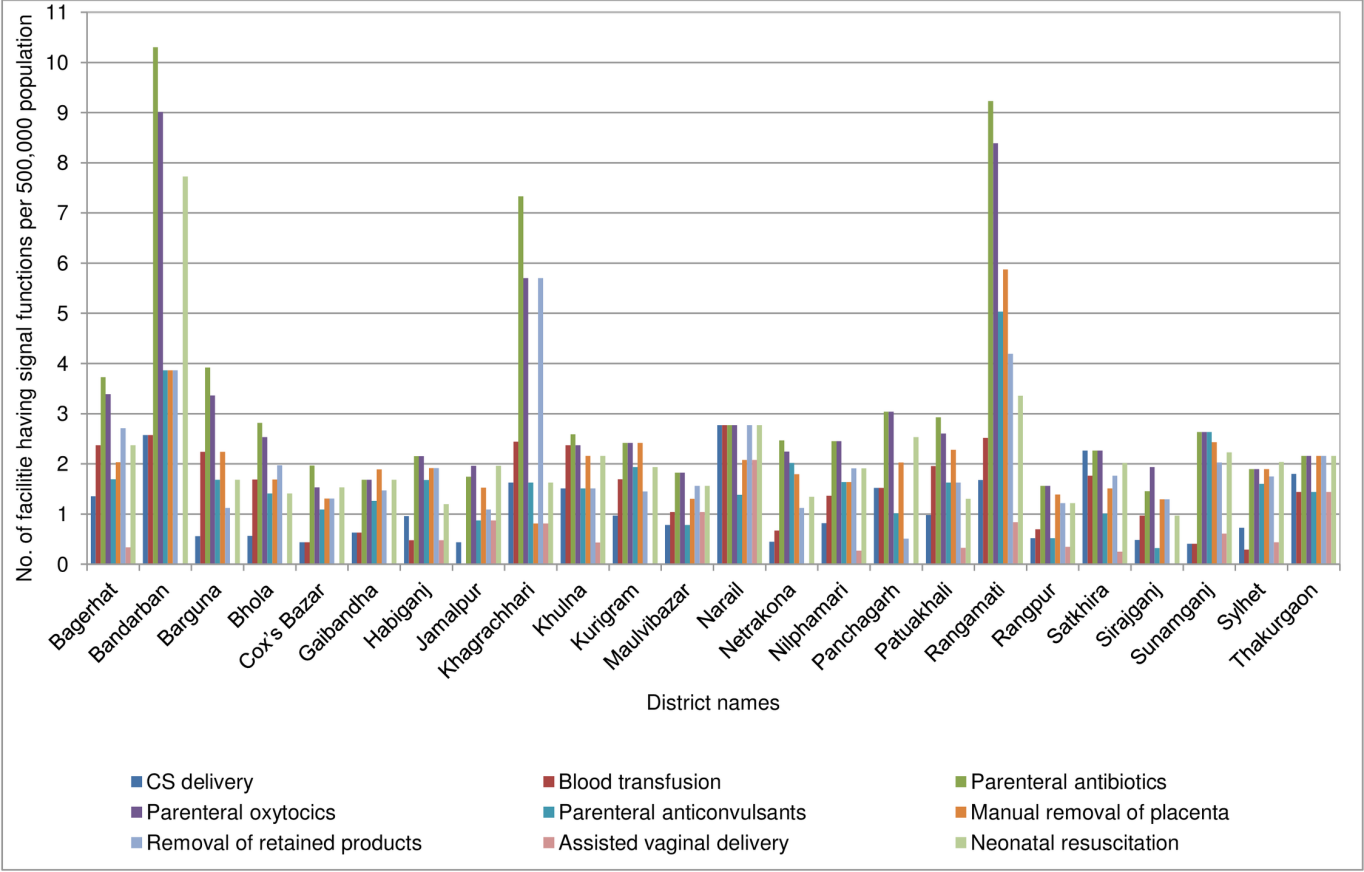
Population coverage of signal functions for EmONC in the public facilities in the last 3 months prior to the date of assessment in the 24 study districts of Bangladesh.

**Fig 3:**
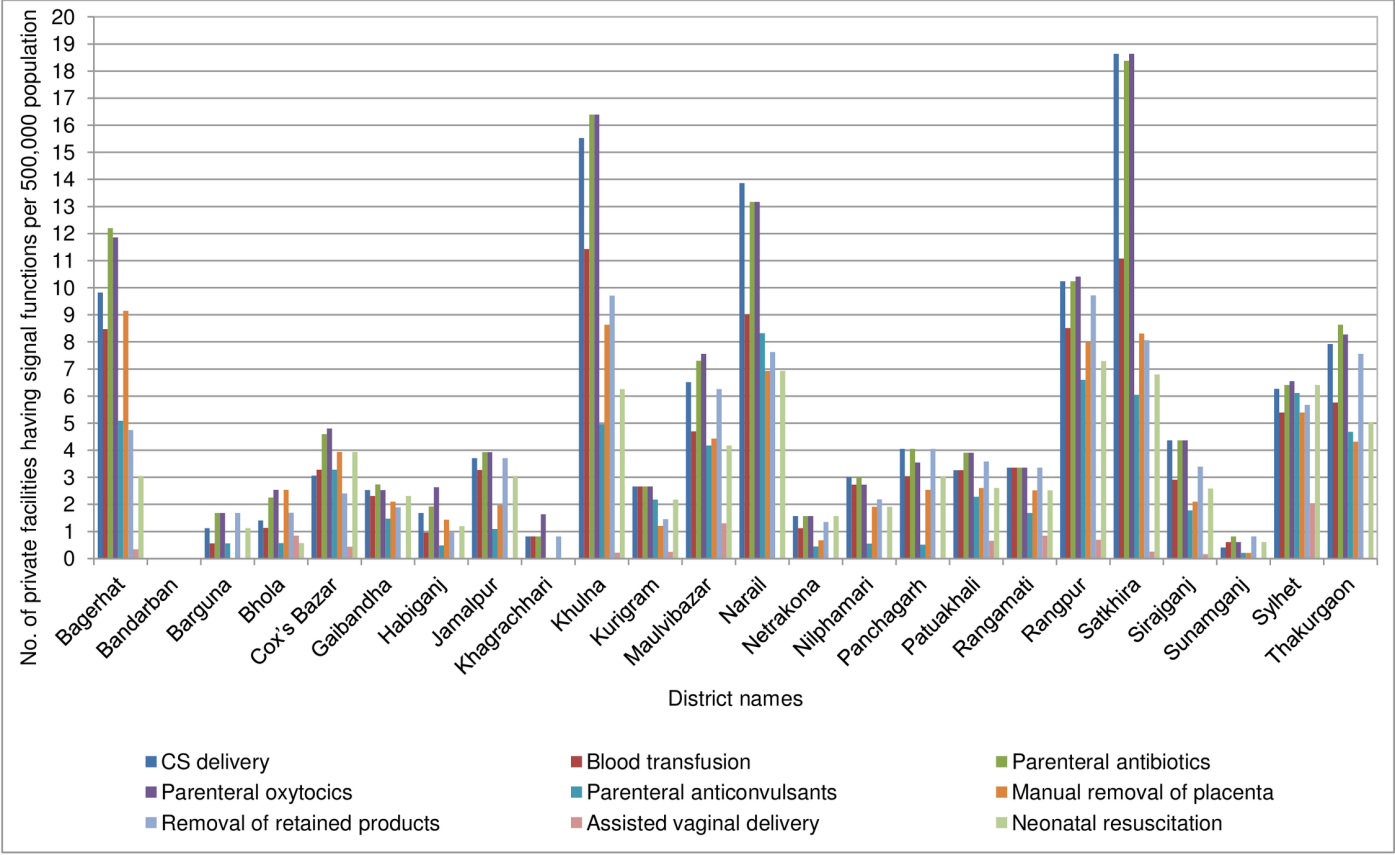
Population coverage of signal functions for EmONC in the private facilities in the last 3 months prior to the date of assessment in the 24 study districts of Bangladesh.

Further examination of data by district revealed major inter-district variations for population coverage of signal functions in each facility of the public and private sectors (Figs 2 and 3). In the public sector, in the majority of the districts (13 of 24), the coverage of either of the CS delivery or BT services was less than 1 per 500,000 population. For the BEmONC signal functions, except for the 3 hill-districts (Bandarban, Khagrachhari and Rangamati), none of the study districts had fulfilled the minimum population coverage for the relevant services. In the private sector, overall the inter-district variation for population coverage of signal function was even greater. While in 5 districts, more than 8 private facilities per 500,000 population provided both CS delivery and BT services, in another 4 districts either of these services were not available in at least 1 facility per 500,000 population.

**Fig 4:**
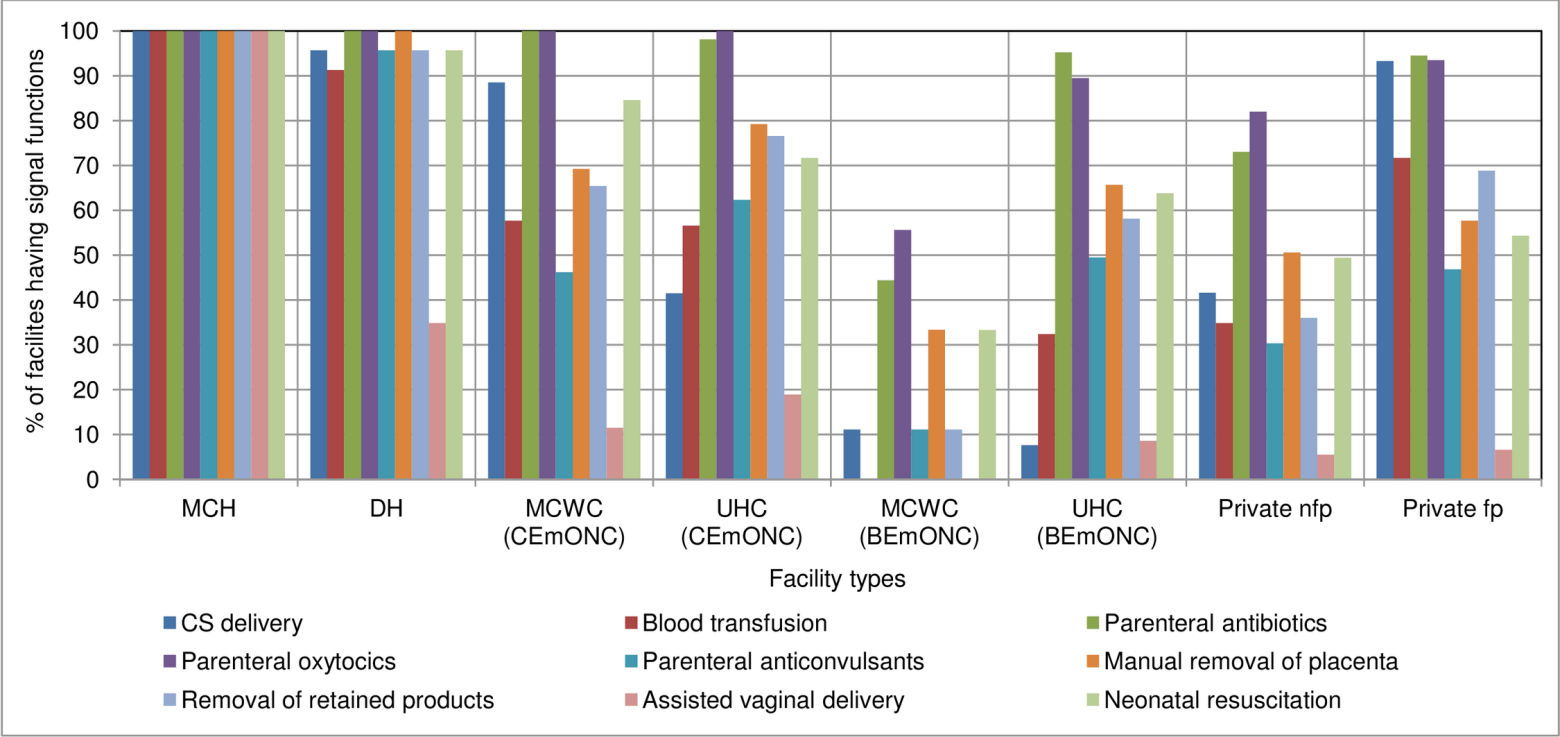
Percentage distribution of availability of signal functions for EmONC by type of facilities in the last 3 months prior to the date of assessment in the 24 study districts in Bangladesh.

Assessment of the service availability by type of health facility reveals that none of the types of facilities, except the public MCHs, had 100% of all the nine signal functions in the last three months prior to the date of assessment (Fig 4). Although over 90% of DHs had CS delivery and BT services, less than 60% of UHCs (CEmONC) that were located in the sub-district level had these services. On the other hand, even though the BEmONC facilities did not require providing CS delivery and BT services, certain proportions of MCWCs and UHCs of this category performed at least one of these services. Again, these services were much less available from private nfp facilities (42% and 35%) than in the private fp facilities (93% and 72%).

Administering parenteral antibiotics and oxytocics was commonly available in all types of public CEmONC facilities. However, in the case of BEmONC facilities, only 33% to 60% of the basic MCWCs and other public facilities for in-patients provided the relevant services, although about 90% to 95% basic UHCs had these services. These two signal functions were also available in about 73% to 94% of the private facilities (both nfp and fp). However, parenteral anticonvulsants that are required to manage eclampsia/preeclampsia were scarce in all types of facilities (11% to 62%), except DHs (96%) and MCHs (100%). Services for both manual removal of placenta and removal of retained products were also not available from a substantial proportion of the facilities, except DHs and MCHs. AVD was the least-available signal function in all types of facilities—even only about 35% DHs provided this service within the last three months prior to the date of assessment.

## Discussion

The study found that the population coverage of CS delivery and BT services in public facilities was close to UN requirement of minimum 1 facility per 500,000 population but, for each of the rest 7 signal functions, the public sector alone could meet the minimum requirement of 5 facilities for the same population. Moreover, there were major inter-district variations in population coverage of signal functions for EmONC in both public and private sectors. Nevertheless, the public and private sectors together could attain the adequate service coverage for 7 out of 9 signal functions (except administration of parenteral anticonvulsants and manual vacuum extraction). In terms of the availability of services in different types of facilities, none, including the private facilities had the required availability for all the signal functions. The only exceptions were the MCHs and DHs. Moreover, at the sub-district level, public facilities had less availability of signal functions than those at the district level.

In our study, the findings of the inter-district variation in the availability of signal functions for EmONC are broadly consistent with the findings of one of our previous studies that reported population coverage of health facilities for obstetric care services [13]. As availability of health facilities does not necessarily ensure availability of services, our current study is unique in reporting the population coverage of signal functions for EmONC in 24 districts of Bangladesh. The towering levels of population coverage of signal functions for EmONC in the private sector are observed in the bordering districts of the south western part of the country; This is related to high concentration of private facilities as noticed previously [13].

Although the availability of most signal functions was higher in the public CEmONC facilities, there was a huge gap in the availability of basic signal functions in the public facilities designated for BEmONC. Albeit designated as CEmONC facilities, about one-third of the public facilities did not have CS delivery services that are necessary to manage obstetric complications. However, most of the private fp facilities had the CS delivery service. Higher availability of CS delivery in private than public facilities gives rise to significant disproportionate results in improving equity of health services for the poor. As per policy of the Bangladesh Government, when public facilities provide services for free of charge, less availability of CS delivery service creates a barrier for poor population in accessing the service. Particularly, when CS delivery is available in less than one public facility designated for CEmONC in contrast to five private facilities per 500,000 populations, it becomes a major concern for the poor. Thus, the private facilities should be taken into consideration for providing services at affordable cost under the direct monitoring and supervision by a national body for increasing the coverage of signal functions for EmONC. At the same time, further research is required to explore the reasons behind high proportion of CS delivery in private facilities by sensitizing the providers on performing CS delivery by properly following the indications in order to avoid unnecessary surgical intervention.

AVD was the least-available service among all the nine signal functions in both public and private sectors. While about three-quarters of the district-level public facilities designated for CEmONC did not provide AVD service, it was further less available both in public BEmONC facilities at the sub-district level and private facilities. Care providers should be trained and encouraged to conduct AVD where indication is appropriate in order to prevent unnecessary CS delivery and related post-surgical complications which can lead to adverse health outcomes for the mothers.

It is clear from this study that the public sector alone is unable to meet the EmONC service requirement throughout the country. A strategy is needed to foster PPP to fill the gaps in the availability of EmONC services nationwide. A mapping of service availability with the help of geographic information system (GIS) application [14] may help identify the gaps and, accordingly, services should be ensured in both public and private sectors, complementing each other’s gaps in areas where needed.

The finding on less availability of signal functions for EmONC in facilities at the sub-district level is consistent with those from the reports of several earlier studies [13,15–16]. Inadequate availability of services at the lower-level facilities eventually results in increased workload in the district-level facilities due to the fact that the cases that could have been managed at the sub-district level would have to go to the district-level facilities because of the unavailability of services at the lower level. As a result, possibility of more adverse outcomes of maternal complications is likely to happen due to delay in reaching the functional facility at the time of emergency [17], especially when an effective referral system is lacking [18]. Structured referral system should be introduced at all levels of the healthcaresystem to save the lives of mothers and the newborns.

### Strengths and limitations of the study

The strength of the study is: it documents both population coverage as well as the availability of signal functions for EmONC. This helps identify the gaps in infrastructure, including the functionality of the existing facilities. However, one limitation of this study is that it did not explore the causes of unavailability of signal functions in different facilities. As documented by other studies, the major reason of unavailability of signal function is the lack of trained human resources in the public sector [13,15]. At the same time, it is important to mention that there is yet no specific policy for enforcing the private-sector facilities to make all the signal functions available to operate as a designated EmONC facility.

## Conclusions

In the public-sector facilities, Bangladesh has a reasonably good coverage of CEmONC services; yet it cannot ensure availability of all the signal functions for EmONC across the country. However, the public and private sector together can ensure most of the signal functions as per UN requirements. Strategic planning is needed to promote PPP for nationwide coverage of EmONC services. Moreover, further studies are needed to understand the reasons behind low availability of the signal functions in public facilities at the sub-district level. Future studies may explore which appropriate initiatives are to be taken for improvement of maternal and newborn care services in these first-level referral facilities.

## Supporting information

S1 Table Data for Table 1. (XLSX)

S2 Table Data for Table 2. (XLSX)

S1 Fig Data for Fig 2. (XLSX)

S2 Fig Data for Fig 3. (XLSX)

S3 Fig Data for Fig 4. (XLSX)
